# Data on three-year monitoring of benthic macroinvertebrates in ditches of the orchard region of Altes Land, Germany

**DOI:** 10.1016/j.dib.2020.106593

**Published:** 2020-11-26

**Authors:** Stefan Lorenz, Angelika Süß

**Affiliations:** Julius-Kuehn-Institute, Institute for Ecological Chemistry, Plant Analysis and Stored Product Protection; Königin-Luise-Straße 19, 14195 Berlin, Germany

**Keywords:** Agriculture, Biodiversity, Environmental monitoring, Freshwater monitoring, Macro invertebrates

## Abstract

The data presented in this article are related to the research article 'Chemical and biological monitoring of the load of plant protection products and of zoocoenoses in ditches of the orchard region Altes Land' (Süß et al., 2006) [1], which is only available in German. The benthic macro invertebrate data presented here were acquired from four ditches (three ditches were located in apple orchards, and one ditch was located in a grassland region) between 2001 and 2003 (Süß & Lorenz, 2020) [2]. This article describes the methods used to record the benthic macro invertebrate species. The field data set is publicly available at the OpenAgrar repository under https://doi.org/10.5073/20201029-170047[2]. It is related to two field data sets, in which pesticide monitoring data (Lorenz et al., 2018) [3] and zooplankton monitoring data (Lorenz & Mueller, 2019) [4] from the same ditches and time period have been presented.

## Specifications Table

**Table:** 

Subject	Environmental science
Specific subject area	Freshwater ecology, ecotoxicology, environmental monitoring
Type of data	Table
How data were acquired	Ditch sediment was removed ∼1 cm deep by a shovel sampler (sampled sediment area: 0.1 m²) in five consecutive replicates. The water body including submersed macrophytes was sampled at a length of 5 m with a dip net (20 × 15 cm opening) in five consecutive replicates.
Data format	Raw
Parameters for data collection	Benthic invertebrate abundances
Description of data collection	Benthic invertebrate species were sampled in agricultural ditches. Three sampled ditches were located in apple orchards, and one ditch was located in a grassland region
Data source location	Orchard region of Altes Land, Germany; ditches in Neuenkirchen, Jork and Estebrügge (2 ditches)
Data accessibility	The field data set is publicly available at the OpenAgrar repository under https://doi.org/10.5073/20201029-170047 https://www.openagrar.de/receive/openagrar_mods_00064486
Related research article	A. Süß, G. Bischoff, A. Mueller, L. Buhr, Chemical and biological monitoring of the load of plant protection products and of zoocoenoses in ditches of theorchard region „Altes Land“, Nachrichtenbl. Deut. Pflanzenschutzd., 58 (2006) 28-42.

## Value of the Data

• The data can be related to previously published data on pesticide contamination generated by either event-driven or weekly integrated sampling.
• The data presented here allow for realistic assessment of effects of simultaneously recorded pesticide concentrations (data linked at the repository) on benthic invertebrate communities.
• The data allow other researchers to perform statistical (meta-)analysis on the impact of agriculture on freshwater ecosystems.

## 1 Data Description

The article presents benthic macro invertebrate data from monitoring in four ditches of the orchard region of ‘Altes Land’, Germany [2]. The data consist of one Excel sheet providing the results of the species determination including ID_ART codes of the taxa [5] to relate to further databases. The benthic macro invertebrate field data set consists of 81.142 species observations and is publicly available at the OpenAgrar repository under https://doi.org/10.5073/20201029-170047 [2]. A total of 165 taxa were recorded (Table 1). The data can be related to data on pesticide analysis with description of the study site and pesticide sampling details [3], which are publicly available at the OpenAgrar repository under https://doi.org/10.5073/20180213-144359 [6]. The data can also be related to zooplankton species data [4], which are publicly available at the OpenAgrar repository under https://doi.org/10.5073/20181213-163305 [7].

**Table 1:** Complete taxa list of the invertebrate data set. Ind. = individuals, ad = adult, l = larvae, juv = juvenile.

Taxa	Ind.	Taxa	Ind.	Taxa	Ind.
**Acari**	**6492**	*Rhantus* sp. (l)	1	Chaoboridae	597
Hydracarina gen. sp.	780	Haliplidae	445	*Chaoborus crystallinus*	585
Oribatei gen. sp.	5712	*Haliplus* sp. (ad)	250	*Chaoborus flavicans*	3
**Araneae**	**9**	*Haliplus* sp. (l)	190	*Chaoborus obscuripes*	9
Araneae gen. sp.	9	*Peltodytes caesus* (ad)	1	Chironomidae gen. sp.	1556
**Bivalvia**	**235**	*Peltodytes caesus* (l)	4	Culicidae	5
Sphaeridae	235	Hydraenidae	2	*Culex* sp.	5
*Musculium lacustre*	50	*Hydraena* sp. (ad)	2	Diptera gen. sp.	126
*Pisidium* sp.	1	Hydrophilidae	51	Dixidae	11
*Sphaerium corneum*	184	*Anacaena limbata* (ad)	5	*Dixella* sp.	11
**Coleoptera**	**1147**	*Enochrus affinis* (ad)	1	Ephydridae	5
Curculionidae	70	*Enochrus quadripunctatus* (ad)	4	Ephydridae gen. sp.	4
*Tanysphyrus lemnae* (ad)	70	*Enochrus* sp. (l)	12	*Setacera* sp.	1
Dytiscidae	311	*Enochrus testaceus* (ad)	1	Sciomyzidae gen. sp.	9
*Acilius canaliculatus* (ad)	1	*Helochares obscurus* (ad)	7	Stratiomyidae	94
*Agabus* sp. (l)	1	*Helochares obscurus* (l)	4	*Odontomyia* sp.	82
*Hygrotus impressopunctatus* (ad)	2	*Helophorus* sp. (ad)	6	*Oplodonta viridula*	7
Colymbetinae gen. sp. (l)	2	*Hydrobius fuscipes* (ad)	2	*Stratiomys* sp.	5
*Dytiscus marginalis* (l)	1	*Hydrobius fuscipes* (l)	1	Tabanidae	4
*Graphoderus cinereus* (ad)	1	*Hydrophilus caraboides* (ad)	1	*Chrysops* sp.	4
*Graptodytes pictus* (ad)	158	*Hydrophilus* sp. (l)	3	Tipulidae	9
*Graptodytes* sp. (l)	30	*Hydrous aterrimus* (ad)	1	*Tipula* sp.	9
*Guinotus pusillus* (ad)	2	*Spercheus emarginatus*	3	**Ephemeroptera**	**6879**
Hydroporinae gen. sp. (l)	18	Noteridae	111	Baetidae	6778
*Hydroporus angustatus* (ad)	2	*Noterus crassicornis* (ad)	110	*Cloeon dipterum*	6778
*Hydroporus palustris* (ad)	10	*Noterus crassicornis* (l)	1	Caenidae	101
*Hygrotus inaequalis*	2	Scirtidae gen. sp. (l)	157	*Caenis robusta*	96
*Hyphydrus ovatus* (ad)	7	**Collembola**	**3603**	*Cloeon dipterum*	5
*Hyphydrus ovatus* (l)	11	Poduridae	3603	**Gastropoda**	**23839**
*Ilybius ater* (ad)	2	*Podura aquatica*	3603	Bithyniidae	256
*Ilybius* sp. (l)	10	**Diptera**	**2635**	*Bithynia leachii*	95
*Laccophilus minutus* (ad)	5	Ceratopogonidae	219	*Bithynia tentaculata*	161
*Laccophilus minutus* (l)	44	*Anopheles* sp.	24	Gastropoda gen. sp.	4
*Rhantus grapii* (ad)	1	Ceratopogonidae gen. sp.	195	Lymnaeidae	6951
*Galba truncatula*	47	*Sigara falleni* (ad)	2	*Helobdella stagnalis*	563
*Lymnaea stagnalis*	875	*Sigara fossarum* (ad)	23	*Theromyzon tessulatum*	264
*Radix ovata*	5251	*Sigara semistriata* (ad)	10	Haemopidae	5
*Stagnicola palustris-complex*	778	*Sigara* sp. (juv)	932	*Placobdella costata*	1
Physidae	8087	*Sigara striata* (ad)	313	*Haemopis sanguisuga*	4
*Physa fontinalis*	8087	Gerridae	6	Piscicolidae	21
Planorbidae	8111	*Gerris* sp. (ad)	2	*Piscicola geometra*	21
*Anisus vortex*	3364	*Gerris* sp. (juv)	4	**Hydrozoa**	**1284**
*Anisus vorticulus*	957	Mesoveliidae	1	Hydridae gen. sp.	1284
*Bathyomphalus contortus*	236	*Mesovelia furcata* (ad)	1	**Isopoda**	**4359**
*Gyraulus albus*	244	Naucoridae	322	Asellidae	4359
*Gyraulus crista*	183	*Ilyocoris cimicoides* (ad)	93	*Asellus aquaticus*	4359
*Hippeutis complanatus*	166	*Ilyocoris cimicoides* (juv)	229	**Lepidoptera**	**279**
*Planorbarius corneus*	1442	Nepidae	1	Pyralidae gen.sp.	279
*Planorbis carinatus*	180	*Nepa cinerea* (juv)	1	**Megaloptera**	**4**
*Planorbis planorbis*	1309	Notonectidae	300	Sialidae	4
*Planorbis planorbis carinatus*	20	*Notonecta glauca* (ad)	50	*Sialis* sp.	4
*Segmentina nitida*	10	*Notonecta* sp. (juv)	250	**Nematoda**	**19**
Valvatidae	301	Pleidae	1144	Nematoda gen. sp.	19
*Valvata cristata*	70	*Plea minutissima* (ad)	540	**Odonata**	**1254**
*Valvata piscinalis*	231	*Plea minutissima* (juv)	604	Aeshnidae	11
Viviparidae	129	Veliidae	74	Aeshnidae gen. sp.	2
*Viviparus contectus*	129	*Microvelia reticulata* (ad)	49	*Anax* sp.	9
**Heteroptera**	**4029**	*Velia* sp. (juv)	25	Coenagrionidae	822
Corixidae	2181	**Hirudinea**	**5001**	Coeagrionidae gen. sp.	51
*Arctocorisa* sp. (juv)	3	Erpobdellidae	2751	*Coenagrion puella/pulchellum*	765
*Callicorixa praeusta* (ad)	3	*Erpobdella nigricollis*	641	*Coenagrion* sp.	1
*Corixa punctata* (ad)	6	*Erpobdella octoculata*	2091	*Ischnura elegans*	5
*Corixa* sp. (juv)	22	*Erpobdella testacea*	19	Gomphidae gen. sp.	1
Corixinae gen. sp. (juv)	626	Glossiphoniidae	2224	Lestidae	12
*Cymatia coleoptrata* (ad)	68	*Glossiphonia complanata*	364	*Chalcolestes viridis*	12
*Cymatia coleoptrata* (juv)	30	*Glossiphonia heteroclita*	944	Libellulidae	5
*Hesperocorixa linnaei* (ad)	103	*Glossiphonia* sp.	21	Libellulidae gen. sp.	4
*Hesperocorixa* sp. (juv)	40	*Hemiclepsis marginata*	68	*Platycnemis pennipes*	2
*Sympetrum vulgatum/sanguineum/ striolatum*	1	*Oecetis furva*	3		
		*Triaenodes bicolor*	442		
Zygoptera gen. sp.	401	**Tricladia**	**2862**		
**Oligochaeta**	**14438**	Dendrocoelidae	289		
Lumbriculidae	59	*Dendrocoelum lacteum*	289		
*Rhynchelmis limosella*	59	Dugesiidae	1058		
Naididae	4797	*Dugesia lugubris / polychroa*	1058		
*Dero digitata*	465	Planariidae	1498		
*Stylaria lacustris*	4332	*Planaria torva*	401		
Oligochaeta gen. sp.	9582	*Polycelis nigra / tenuis*	1097		
**Ostracoda**	**2311**	Tricladida gen. sp.	1		
Ostracoda gen. sp.	2311	Typhloplanidae	16		
**Trichoptera**	**463**	*Bothromesostoma personatum*	5		
Leptoceridae	463	*Mesostoma ehrenbergii*	9		
*Athripsodes aterrimus*	18	*Mesostoma tetragonum*	2		

## 2 Experimental Design, Materials and Methods

Species composition and species abundances of the benthic macro invertebrate communities were analysed in four ditches located in agricultural land. These sites were selected on the basis of the expected differences in their pesticide exposure. Two ditches were located in apple orchards managed by integrated pest management (distance to the sprayed trees: 2.9 m for the ditch “Neuenkirchen”, 4.5 m for the ditch “Estebrügge”). The ditch “Jork” was located within an ecologically managed apple orchard with a distance of 5.8 m to the sprayed trees. These three ditches were monitored from 2001 to 2003. The ditch “Estebrügge-Grassland”, monitored from 2002 to 2003, served as an additional reference site as it was not located in pesticide treated plantations. Please see Lorenz et al. (2018) [3] for further study site characteristics (Table 3 adopted from [1]).

Seven monthly samplings were performed in 2001 (April to October) and four samplings in April, June, July, and September in 2002 and 2003. At each sampling date, ditch sediment was removed approximately 1 cm deep by a shovel sampler (sampled sediment area: 0.1 m²). This was done in five consecutive replicates with a distance of 20 m between them, resulting in a total sampled area of total: 0.5 m². Similarly, the water body including submersed macrophytes was sampled at a length of 5 m with a dip net (20 × 15 cm opening) in five consecutive replicates with a distance of 20 m between them. This resulted in a total sampled water volume of 750 L equaling a sampled ditch area of 5 m². The samples were washed in the laboratory using a sieve with 0.5 mm mesh size and fixed in 70% alcohol. The specimens were determined to the lowest taxonomic level whenever possible, only Aranea, Nematoda, Ostracoda, Hydracarina, and Oribatei remained on their higher taxonomic levels.

## CRediT Author Statement

**Stefan Lorenz**: Conceptualization, Formal analysis, Investigation, Writing - original draft, Writing - review & editing, Visualization. **Angelika Süß**: Methodology, Investigation, Writing - review & editing.

## Declaration of Competing Interest

The authors declare that they have no known competing financial interests or personal relationships which have or could be perceived to have influenced the work reported in this article.
